# Increasing surgical healthcare utilization for infants with congenital anomalies in Texas

**DOI:** 10.3389/fsurg.2025.1620628

**Published:** 2025-07-16

**Authors:** Bea B. Jeon, Sarah Peiffer, Shannon M. Larabee, Kathleen Hosek, Dailen Alonso, Timothy C. Lee, Sundeep G. Keswani, Alice King

**Affiliations:** ^1^ Michael E. DeBakey Department of Surgery, Baylor College of Medicine, Houston, TX, United States; ^2^ Department of Surgery, Division of Pediatric Surgery, Texas Children’s Hospital, Houston, TX, United States

**Keywords:** congenital anomalies (CAs), burden of care, cost of care, charges of care, infant surgical care, length of stay (LOS), children's surgery verification (CSV), disability-adjusted life years (DALY)

## Abstract

**Introduction:**

Congenital anomalies (CAs) impact 3% of live births and account for disproportionately high healthcare costs. While many CAs require multidisciplinary care and surgical intervention, the overall financial impact of infants diagnosed with CA with surgical needs is unknown. We aim to evaluate and characterize the charges of care in infants with CA and surgical needs in Texas.

**Methods:**

A database study using the Texas Inpatient Public Use Data File was performed to query infants (<365 days) statewide from 1/2021 to 12/2021 for admissions with CA and involved organ system by ICD-10 codes. Encounters transferred to an outside hospital were excluded to avoid systematic double-counting. Descriptive statistics were performed.

**Results:**

Of 376,215 total admissions, 81,666 had surgical needs with OR charges. While non-CA represent the majority of surgical admissions (63,895/81,666; 78.24%), CA-surgical admissions represent 73.3% ($4.766/$6.496 billion) surgical admissions charges. Of CA-surgical admissions, 78.9% were single organ-system (1CA) with 14.5% with two organ-systems (2CA), 4.0% with three organ-system (3CA) and 2.6% with 4 + organ-systems (4 + CA). The proportion of admissions with surgical needs increases with the number of CA organ-systems involved. The median charge per CA-surgical admission was $1,296 for1CA, $4,517 for 2CA, $20,272 for 3 CA, and $25,313 for 4 + CA compared to the $797 for non-CA surgical admissions. Surgical admission charges increase with the number of CA organ-systems involved.

**Conclusions:**

Surgical care of CA in infants is associated with significant healthcare utilization, accounting for $4.8 billion (73.4%) of all inpatient charges in 2021 despite representing a minority of admissions. Increasing number of CA organ-systems involved is associated with an increased proportion of patients with surgical admissions and increased median charge of admission.

## Introduction

Congenital anomalies (CAs), also known as birth defects, are structural or functional abnormalities that can result from environmental teratogens, nutritional deficiencies, gene defects, chromosomal disorders, and other factors during gestational development. Common CAs include cleft lip/palate, heart defects, atypical limbs, neural tube defects, chromosomal syndromes, metabolic disorders, and degenerative disorders (1). CAs impact 3% of live births in the US and is the highest cause of infant deaths at 20% in 2021 (2).

CAs can vary in interventions, morbidity, and mortality rates based on type of CA, individual severities, and pathologies. While Congenital Diaphragmatic Hernias have a 1-year survival rate of 64.1% (3), spina bifida has a 92.9% 1-year survival rate (4), cleft lips/palates have a 98% 1-year survival rate, and gastroschisis with a 93% 1-year survival rate (5). Similarly, interventions to treat CA vary broadly. While biliary atresia uniformly requires surgical intervention (6), congenital heart disease may be monitored or treated with a combination of catheter-based, medication, and/or surgical intervention (7). The type and severity of CA determines the extent of pharmaceutical, cosmetic, medical, and/or surgical attention to address functionality, quality of life, and aesthetic needs.

Many CA interventions require multidisciplinary or sub-specialized treatment. In efforts to ensure optimal resource allocation and clinical outcomes in surgical procedures, the American College of Surgeons (ACS) has created the Children's Surgery Verification (CSV) Quality Improvement Program with certification to identify institutions with resources equipped to manage complex pediatric surgical care (8). It is important to recognize that not all CA patients requiring surgical interventions benefit or should be expected to be treated at CSV centers. While CSV centers have been demonstrated to have improved outcomes in some select populations (9), further investigation is needed to determine the impact of CSV institutions on the cost of care of CAs.

Furthermore, CAs account for disproportionately high healthcare costs in the US as treatments are costly and can extend past infancy through lifelong complications. US Patients with any CA were reported to have about double the mean hospitalization costs throughout their life compared to patients with no anomalies (10). Patients with CAs may require not only surgical interventions but also other inpatient and outpatient care, rehabilitation, medication, neonatal intensive care unit stays, etc. Additionally, CA patients often have longer lengths of stay (LOR) in hospital, with an average of 18 days in the hospital in their first year of life, compared to the two days from children without anomalies (11). The LOS also may increase with the type and severity of anomaly (e.g., neonates with small intestine atresia had an average length of stay of 92 days in the first year of life compared to 28 days for neonates with Down syndrome). These additional medical demands and the resulting financial costs place a higher burden of care for patients and families. However, while many CAs require multidisciplinary care and surgical intervention, the overall financial impact of infants diagnosed with CA with surgical needs is unknown. We aim to evaluate and characterize the care of infants with CA and surgical needs in Texas.

## Methods

We performed a retrospective database study using the Texas Inpatient Public Use Data File (TIPUDF), a statewide hospital discharge database (Texas Department of State Health Services & Center for Health Statistics, 2021). This data is reported annually by quarter. Baylor College of Medicine granted an Institutional Review Board exemption as this data is publicly available and de-identified. Analysis and reporting were conducted per the Strengthening the Reporting and Observational Studies in Epidemiology guidelines (12).

We conducted a database query for Texas infants (<365 days) statewide from 1/2021 to 12/2021 for all inpatient admissions (Supplementary File). Encounters transferred to an outside hospital were excluded to avoid systematic double-counting.

### Variables

Data extraction included type of length of stay, type of admitting facility, type and source of admission, CA diagnosis codes, illness severity score, discharge outcomes, OR occurrence, and charges. CVS centers were identified through institution name. International Classification of Disease, Tenth Edition (ICD-10) diagnostic codes were used to identify diagnosis of CA and categorize organ systems involved for purpose of analysis (Supplementary Table 1). Organ systems were categorized by number of organ systems involved.

### Outcome

Primary study outcome was the charges of inpatient admissions and healthcare resources utilization in the care for neonates and infants with CAs in Texas. Healthcare resource utilization was evaluated as number of inpatient admissions, transfers, CSV institutional care, severity, length of stay, and mortality.

### Statistical analysis

Descriptive statistics are performed. Categorical variables are presented as a number (percent) while continuous variables are presented as median with interquartile range (IQR) for admission level data. Bivariate analysis was performed using Pearson's Chi-squared test for categorical variables and Kruskal–Wallis test for ordinal variables. Analysis was performed using SAS version 9.4 (SAS Institute, Cary, NC).

## Results

### Total cohort demographics

In the 2021 Texas Inpatient Public Use Data File there were 381,257 total admissions, and 5,042 (1%) encounters transferred out were excluded to avoid double counting. Therefore, our cohort of 376,215 admissions were analyzed, and 81,666 had surgical needs with OR charges. 48.5% (182,303/376,215) of patients were female, and 0.1% (457) was unidentified or missing. The ethnic demographics of our cohort was 63.7% (239,533) white, 12.2% (46,046) Black, 4.6% (17,336) Asian, 0.2% (933) Native American, 19.2% (72,358) Other, and 9 were missing or invalid categorization. 36.1% (135,650) were Hispanic. Most patients (333,379, 90.1%) were from urban areas, while only 9.3% 934,002) were from rural areas. CA types and number of patients included in this study can be seen in Table 1.

**Table 1:** Distribution across organ systems involved in congenital anomalies.

CA organ system	*n*	(%)
Nervous system anomaly	2,123	2.74
Face anomaly	12,759	16.45
Circulatory anomaly	17,220	22.20
Respiratory anomaly	1,494	1.93
Digestive anomaly	1,777	2.29
Genital anomaly	5,743	7.40
Urinary anomaly	3,466	4.47
Musculoskeletal anomaly	6,671	8.60
Other anomaly	23,684	30.54
Chromosomal anomaly	2,619	3.38
Total^a^	77,556	

^a^ Patients with more than 1 CA are counted towards multiple categories and the total does not add up to total CA patients.

### Total cohort charges by CA

17.1% (64,509/376,215) of all admissions were CA patients, and 15.2% (9,787/64,509) of CA patients, or 2.6% (9,787/376,215) of all patients, had more than one type of organ system involved. CA patients were a minority of all admissions but represented 55% (7.336/13.345 billion) of total charge of admissions and 73.4% (4.766/6.496 billion) of total charges of admissions with OR charges. Overall, as the number of CA systems increased, the median charge per admission increased. Non-CA patients had a median charge of $5,142.3 [3,591.8, 8,818], 1 CA patients had a median charge of $6,923.8 [4,171.1, 25,418.6], 2 CA patients had a median charge $17,836.9 [5,411.2, 131,264.5], 3 CA patients had a median charge of $119,263.5 [25,570.1, 468,972.0)] and 4 + patients had a median charge of $290,148.0 [90,955.0, 727, 649.0] per admission.

### Total cohort admission source, severity, and outcomes by CA

The increasing number of CAs is also associated with an increase in transfers, emergent cases, surgical needs, length of stay, illness severity, and mortality. Patients were more likely to be admitted through transfer or emergency as the number of organ systems involved in the CA increased (Table 2). The majority (71.4%) of non-CA patients had a severity score of 1(minor) while patients with any CA were more likely to be categorized into a severity score of 2 (moderate) or higher (Table 3). Patients with three or more CA organ systems were more likely to receive a severity score of 3 (major) or 4 (extreme).

**Table 2:** Total cohort admission source by organ systems involved in CA.

Number of CA organ systems involved (*n*)	Inborn, *n* (%)^a^	Transfer, *n* (%)^a^	Emergency, *n* (%)^a^
Non-CA (*N* = 311,706)	288,756 (92.6%)	3,730 (1.2%)	13,627 (4.4%)
1 CA organ systems (*N* = 54,722)	48,202 (88.1%)	1,800 (3.3%)	2,601 (4.8%)
2 CA organ systems (*N* = 7,620)	5,787 (75.9%)	586 (7.7%)	606 (8.0%)
3 CA organ systems (*N* = 1,438)	807 (56.1%)	248 (17.2%)	176 (12.2%)
4+ CA organ systems (*N* = 729)	333 (45.7%)	213 (29.2%)	87 (11.9%)
Total (*N* = 376,215)	343,885 (91.4%)	6,577 (1.7%)	17,097 (4.5%)

^a^ Chi-Square *p*-value of <0.001.

**Table 3:** Total cohort LOS, severity, and mortality by increasing CA organ system.

Number of CA organ systems involved (*n*)	Length of stay days [IQR]^b^	APR-DRG Severity of Illness Score*^,^^a^				Mortality *n* (%)^a^
		1	2	3	4	
Non-CA (*N* = 311,706)	2.0 (1.0, 2.0)	222,468 (71.4%)	64,951 (20.8%)	22,777 (7.3%)	1,412 (0.5%)	636 (0.2%)
1 CA organ systems (*N* = 54,722)	2.0 (2.0, 3.0)	26,822 (49.0%)	17,935 (32.8%)	8,051 (14.7%)	1,853 (3.4%)	334 (0.6%)
2 CA organ systems (*N* = 7,620)	3.0 (2.0, 12.0)	2,206 (29.0%)	2,724 (35.7%)	1,843 (24.2%)	826 (10.8%)	154 (2.0%)
3 CA organ systems (*N* = 1,438)	10.0 (3.0, 38.0)	149 (10.4%)	435 (30.3%)	494 (34.4%)	350 (24.3%)	77 (5.4%)
4+ CA organ systems (*N* = 729)	23.0 (7.0, 49.0)	9 (1.2%)	157 (21.5%)	293 (40.2%)	269 (36.9%)	107 (14.7%)
Total (*N* = 376,215)	2.0 (1.0, 3.0)	251,654 (66.9%)	86,202 (22.9%)	33,458 (8.9%)	4,710 (1.3%)	1,308 (0.3%)

* Assignment of severity of illness score from the All Patient Refined (APR) Diagnosis Related Group (DRG) from the 3M APR-DRG Grouper. 1, minor; 2, moderate; 3, major; 4, extreme.

^a^ Chi-Square *p*-value of <0.001.

^b^ Kruskal–Wallis *p*-value of <0.001.

### Operative CA and charge

CA patients were significantly more likely to receive surgery during inpatients stays as the number of organ systems increased. 20.5% (63,895/311,706) of non-CA patients were surgical, while 25.6% (14,027/54,722) of 1 CA, 33.7% (2,569/7,620) of 2 CA, 49.7% (715/1,438) of 3 CA, and 63.1% (460/729) of 4+ CA patients were surgical.

The surgical patient cohort had the most distinct charge disparities between the different numbers of CA organ systems involved. While non-CA represents the majority of surgical admissions (63,895/81,666; 78.24%), CA-surgical admissions represent 73.3% ($4.766/$6.496 billion) of surgical admission charges.

Of CA-surgical admissions, 78.9% (14,027/17,771) were single organ-system (1CA) with 14.5% (2,569/17,771) with two organ-systems (2CA), 4.0% (715/17,771) with three organ-system (3CA) and 2.6% (460/17,771) with 4 + organ-systems (4 + CA). The proportion of admissions with surgical needs increases with the number of CA organ-systems involved. Median charges per CA-surgical admission are more than non-CA surgical admissions. Surgical admission charges increased with the number of CA organ-systems involved (Table 4). Additionally, an increase in the number of CAs was associated with an increase in just isolated OR charges. Non-CA patients had a median OR charge of $797.0 [593.0, 2,196.0], 1 CA $1,396.0 [635.0, 4,935.0], 2 CA $4,517.0 [809.0, 29,266.0], 3 CA $20,272.0 [3,792.0, 46,430.0], and 4+ CA $25,313.0 [12,806.4, 47,078.5].

**Table 4:** Surgical cohort charges by increasing CA organ systems.

Admission by number of CA organ system (*n*)	Admissions with OR charge (% Organ system cohort)^a^	Total cost for admissions with OR charge	Median charge per admission with OR charge [IQR]^b^
Non-CA (*N* = 311,706)	63,895 (20.5%)	$ 1,730,346,662	$6,542.8 [4,613.3–10,641.0]
1 CA organ systems (*N* = 54,722)	14,027 (25.6%)	$ 2,652,357,754	$11,435.0 [5,650.3–100,739.0]
2 CA organ systems (*N* = 7,620)	2,569 (33.7%)	$ 1,172,906,407	$110,970.0 [12,472.2–492,660.0 8]
3 CA organ systems (*N* = 1,438)	715 (49.7%)	$ 560,227,483	$350,887.0 [107,118.0–935,910.0]
4+ CA organ systems (*N* = 729)	460 (63.1%)	$ 380,393,191	$520,900.5 [231,765.0–10,66,749.0]

^a^ Chi-Square *p*-value of <0.001.

^b^ Kruskal–Wallis *p*-value of <0.001.

### OP cohort admission source and outcomes by CA

The resulting data reflected an increase in the number of transfers for patients with increased CA systems (Table 5). Only 0.9% of non-CA patients were transferred to a different institution for care, while 6% of 1 CA, 13.7% of 2 CA, and 24.8% of 3 CA, and 34.8% of 4+ CA were transferred. Our results also showed the number of emergency cases increased as the number of CA systems increased. 1.8% (1,179) of non-CA surgical patients were emergent, while 5.8% (816) of 1 CA surgical, 7.5% (193) of 2 CA surgical, 8.8% (63) of 3 CA surgical, and 8.0% (37) of 4+ CA surgical were emergent.

**Table 5:** Surgical cohort by admission type, CSV, and mortality.

Admission Type by number of CA organ system (OR) (*n*)	Transfer *n* (%)^a^	Emergency *n* (%)^a^	CSV *n* (%)^a^	Mortality *n* (%)^a^
No CA (*n* = 63,895)	591 (0.9%)	1,179 (1.8%)	2,159 (3.4%)	148 (0.2%)
1 CA organ system (*n* = 14,027)	843 (6.0%)	816 (5.8%)	1,880 (13.4%)	167 (1.2%)
2 CA organ systems (*n* = 2,569)	353 (13.7%)	193 (7.5%)	688 (26.8%)	72 (2.8%)
3 CA organ systems (*n* = 715)	177 (24.8%)	63 (8.8%)	309 (43.2%)	41 (5.7%)
4+ CA organ systems (*n* = 460)	160 (34.8%)	37 (8.0%)	229 (49.8%)	60 (13.0%)
Total (*N* = 81,666)	2,124 (2.6%)	2,288 (2.8%)	5,265 (6.4%)	488 (0.6%)

^a^ Chi-Square *p*-value of <0.001.

Despite the significantly higher median charge per admission and increased use of medical resources, CA patients risk higher mortality rates compared to non-CA patients. Additionally, an increasing number of CA systems involved is associated with an increased risk of mortality.

### OP cohort CSV institutional care by CA

There was a similar trend of increasing surgical patients receiving care at a CSV certified institution as the number of organ systems with CAs increased. 3.4% of non-CA patients received care at CSV, while 13.4% 1 CA, and 26.8% 2 CA, 43.2% 3 CA, and 49.8% of 4+ CA patients were given care at a CSV institution (Table 5).

## Discussion

### Charge comparison

The results of our study showed that congenital anomalies are associated with higher total and median charges, representing a significant portion of care despite representing a minority of patients. Our data corresponds with the recent 2023 Swanson publication (10) which also found that CA patients incur 7.7% of total inpatient charges despite representing only 4.1% hospitalizations. We also found increasing the number of CA organ-systems involved was associated with an increased proportion of surgical admissions and increased median charges of admission. Non-CA patients made up 78.2% (63,895/17,771) of admissions with OR charges but only 13.3% (1.730/12.992 billion) of total charges for admissions with OR charges.

### Single vs. multiple congenital anomalies

While the total charge for OR admissions was higher was 1CA compared to 4 + CA patients, 1CA patient made up 17.2% of the total OR admissions while 4 + CA patients made up only 0.6% of the total OR admissions. We can further see the increasing individual medical burden as the median charge per OR admission increases with increasing CA. The median charge of 1CA admission is 1.75 times, 2CA is 5.67 times, 3CA is 25.44 times, and 4 + CA charge is 31.76 times the median charge of non-CA admissions. Our results align with the current literature which also found that isolated congenital anomalies incurred less charges compared to multiple comorbid anomalies (13, 14). The increased charges and admissions of CAs may be attributed not only to the need for a multidisciplinary team but also to higher operating costs, transfers, emergency cases, longer lengths of stay, complications, other staff, rehabilitation needs, and surgical re-interventions. The medical burden of care increases drastically as the number of CAs increases, emphasizing the need for further studies of the overall charges and cost of CAs.

### Long term perspective

The first year of life of patients with CAs represents a significant burden to population/health care system (10) and is a crucial period to prevent further disability-adjusted life years (DALYs) (15). While our study provides further insight on the burden of care for CA infants, the 2021 TIPUDF utilized for our study does not allow us to track patients longitudinally as it focuses on infant (<365 days) patients. CA patients continue to require care after the infant period. A 2022 European study found that most CA patients that received surgery had a median of 2 [1.98–2.02] surgical procedures before 5 years of age (16). CA patients that survive infancy may continue to require medical attention for an indefinite period with a potential for lifelong complications and costs. Further studies are required to understand the population-wide, amplified burden of care throughout the lives of CA patients.

Additionally, this study also does not include outpatient care or any other financial, emotional, or physical burdens. Patient families shoulder the grief, emotional support, home patient care, communication with practitioners, education, etc. of having a child with CA (17). Although our study can provide insight on the financial charges of inpatient admissions in the first year of life, further analysis of all medical care and additional burdens is necessary to identify the total financial and emotional burdens.

### Heterogeneity of CAs

Additionally, this study focuses on the presence of one or more systems with CAs. However, CAs are a heterogeneous population with varying medical needs and outcomes. A 2016 paper stated that 23.6% trisomy 13 patients and 13.8% trisomy 18 patients required surgery, and many of the surgical interventions addressed other comorbid anomalies such as congenital heart disease (18). In comparison, 36% of omphalocele patients require surgery to repair the bowl protrusion, with varying urgency and surgical complexity depending on the extent of the protrusion and organs involved (19). Even within the same type of CA, there is a large variation in surgical intervention needs. 90.1% of complex cardiac lesions, such as hypoplastic left heart syndrome, receive interventions while 4.9% of less severe cardiac lesions, such as atrial septal defects, received cardiac interventions (20). The severity of CAs, in addition to the number of overall CA systems involved, determines the extent of surgical interventions required for each patient. Therefore, it is important to recognize the range of severity and resulting medical burden for CA patients and families.

### CSV care

While transfer and emergency are methods of admissions, it indicates a more severe hospitalization than the “inborn” type of admission. Our results indicate that patients with CA are more likely to have higher severity and experience medical complications that require more interdisciplinary and surgical resources not available in the average neonatal department or even the birth institution. Therefore, it is unsurprising to see the increase in transfers and emergency cases as the number of CA systems increases.

Additionally, an increasing number of CAs is associated with higher severity, emergency risk, and transfer needs for surgical CA patients. Institutions transferring patients with such high medical needs imply that the patients cannot receive adequate care and resources at their original institution and require care from specialized centers such as CSV institutions. The transfer process for patients requiring transfer to institutions with more adequate resources such as CSV centers creates a larger burden of care as transportation, housing, and other transfer related costs compound with medical costs. By virtue of the size of the state, heterogeneous population and co-localization of the five Level 1 CSV institutions in three metropolitan areas, disparities likely exist in access to care at CSV centers. Future analysis is required to better understand the benefits and costs of CSV and non-CSV care for different kinds of CA patient needs.

Although the CSV program is a certification and not a replacement standard of care, it allows patients and referring providers to seek care at an institution verified with prespecified resources and standards. Beyond pediatric providers, CSV certification may be helpful in obstetrics and maternal-fetal management of prenatally diagnosed surgical fetal anomalies, which may benefit from early care at centers equipped for neonatal surgery. While not all CA patients require surgical interventions, severe structural anomalies often need surgery during the neonate period. Of the CA patients requiring surgical intervention, not all require care at CSV certified institutions.

We currently do not have a standard of specific characteristics to determine the need to transfer care to CSV institutions. Our results indicate an increase in CA systems is associated with increasing severity levels designated by the All Patient Refined (APR) Diagnosis Related Group (DRG). However, our results also indicate that only some CA patients were transferred to higher care facilities. While higher severity patients were more likely to be transferred to higher care facilities, not all level 3 or 4 severity patients required transfer. Further investigation is required to understand the capacity of non-CSV and CSV institutions to treat and support various severities of CAs. Further specification on the severity and characteristics of transfer requirements to high care facilities such as CSV institutions could better guide local, non-CSV hospitals to determine the best institution for each patient.

### Limitations

The THCIC database only reports data on charges which can vary by hospital and may not be a direct correlation with cost. Although this study tracks the general financial trend of inpatient charges based on number of involved CA organ systems in patient's first year of life, we cannot account for all factors in burdens of care including ambulatory/outpatient and homecare costs. The THCIC is a public database, and patient identifiers have been modified or removed. Due to the lack of patient identifiers, we removed patients that were transferred to outside hospitals. Therefore, our cohort may underestimate the number of CA patients as out-of-state transfers were excluded. Additionally, as this data is sourced from billing, we are unable to avoid possible clerical entry orders for diagnosis, as well as unable to track patients longitudinally through due to the lack of patient identifiers. Hence, some admissions may represent readmissions of individual patients. Prospective, disease specific studies are needed to better delineate the impact of surgical CA diagnoses on healthcare costs and burden.

The Texas Health Care Information Collection (THCIC) database used in this study is collected from the heterogeneous Texas population. Additionally, our study utilizes a large 2021 sample size of 376,215 total admissions and 81,666 had surgical needs with OR charges. Therefore, the significantly higher healthcare charges for CA patients relative to non-CA patients and the increasing charges as the number of CA systems involved may be generalizable to admissions outside of Texas. However, a study including data from multiple states could better represent the national trends for other, non-charge related CA admission data as frequency of births and other factors may not be the same in each state.

## Conclusions

Surgical care of CA in infants is associated with significant healthcare charges, accounting for $4.8 (73.4%) billion in charges in 2021 despite representing a minority of admissions. The increasing number of CA organ-systems involved is associated with an increased proportion of patients with surgical admissions and increased median charge of admission.

Recognizing the burden of care for CAs is essential to provide patients and families with adequate insight and planning. Families are able to prepare for the stressors of supporting their child with comprehensive counseling.

## Data Availability

The datasets presented in this study can be found in online repositories. The names of the repository/repositories and accession number(s) can be found below: https://www.dshs.texas.gov/center-health-statistics/texas-health-care-information-collection/health-data-researcher-information/texas-hospital-emergency-department-research-data-file-ed-rdf/texas-inpatient-public-use-data-file-pudf. Further inquiries can be directed to the corresponding author.
