# Supplementary material for: Increasing surgical healthcare utilization for infants with congenital anomalies in Texas
Source: Front Surg. 2025 Jul 16;12:1620628. doi: 10.3389/fsurg.2025.1620628 (PMC12309273; doi:10.3389/fsurg.2025.1620628)
Supplement: Supplementary file 1 [file Table1.docx]

**Online Only Supplements Contents:**

**Supplemental Table 1: Congenital Anomaly International Classification of Diseases (ICD)-10 Codes and Systems-Based Categories**

**Supplemental Table 1: Congenital Anomaly International Classification of Diseases (ICD)-10 Codes and Systems-Based Categories**

| **Diagnosis** | **ICD-10** |
| --- | --- |
| Congenital Malformations of the Nervous System | Q000, Q001, Q002, Q010, Q011, Q012, Q018, Q019, Q02, Q030, Q031, Q038, Q039, Q040, Q042, Q043, Q044, Q045, Q046, Q048, Q049, Q050, Q051, Q052, Q053, Q054, Q055, Q056, Q057, Q058, Q059, Q060, Q061, Q062, Q063, Q064, Q068, Q069, Q0700, Q0701, Q0702, Q0703, Q078, Q079 |
| Congenital Malformations of the Eye, Ear, Face and Neck (Including Cleft Lip/Palate) | Q100, Q101, Q102, Q103, Q104, Q105, Q106, Q107, Q111, Q112, Q120, Q121, Q122, Q123, Q128, Q130, Q131, Q132, Q133, Q134, Q135, Q1381, Q1389, Q139, Q140, Q141, Q142, Q143, Q148, Q150, Q158, Q159, Q160, Q161, Q162, Q163, Q164, Q165, Q169, Q170, Q171, Q172, Q173, Q174, Q175, Q178, Q179, Q180, Q181, Q182, Q183, Q184, Q185, Q186, Q187, Q188, Q189, Q351, Q353, Q355, Q357, Q359, Q360, Q361, Q369, Q370, Q371, Q372, Q373, Q374, Q375, Q378, Q379 |
| Congenital Malformations of the Circulatory System | Q200, Q201, Q202, Q203, Q204, Q205, Q206, Q208, Q209, Q210, Q211, Q212, Q213, Q214, Q218, Q219, Q220, Q221, Q222, Q223, Q224, Q225, Q226, Q228, Q229, Q230, Q231, Q232, Q233, Q234, Q238, Q239, Q240, Q241, Q242, Q243, Q244, Q245, Q246, Q248, Q249, Q250, Q251, Q252, Q2521, Q2529, Q253, Q254, Q2540, Q2541, Q2542, Q2543, Q2544, Q2545, Q2546, Q2547, Q2548, Q2549, Q255, Q256, Q2571, Q2572, Q2579, Q258, Q259, Q260, Q261, Q262, Q263, Q264, Q265, Q266, Q268, Q269, Q270, Q271, Q272, Q2730, Q2731, Q2732, Q2733, Q2739, Q278, Q279, Q281, Q282, Q283, Q288, Q289 |
| Congenital Malformations of the Respiratory System | Q300, Q301, Q302, Q303, Q308, Q309, Q310, Q311, Q312, Q313, Q315, Q318, Q319, Q320, Q321, Q322, Q323, Q324, Q330, Q331, Q332, Q333, Q334, Q336, Q338, Q339, Q341, Q348, Q349 |
| Other Congenital Malformations of the Digestive System | Q380, Q381, Q382, Q383, Q384, Q385, Q386, Q387, Q388, Q390, Q391, Q392, Q393, Q394, Q395, Q396, Q398, Q399, Q400, Q401, Q402, Q403, Q408, Q409, Q410, Q411, Q412, Q418, Q419, Q420, Q421, Q422, Q423, Q428, Q429, Q430, Q431, Q432, Q433, Q434, Q435, Q436, Q437, Q438, Q439, Q440, Q441, Q442, Q443, Q444, Q445, Q446, Q447, Q450, Q451, Q452, Q453, Q458, Q459 |
| Congenital Malformations of the Genital Organs | Q5001, Q5002, Q501, Q502, Q5032, Q5039, Q504, Q505, Q506, Q510, Q5110, Q512, Q5120, Q5128, Q513, Q514, Q516, Q51810, Q51818, Q51820, Q51828, Q519, Q520, Q5210, Q52129, Q522, Q523, Q524, Q525, Q526, Q5270, Q5271, Q5279, Q528, Q529, Q5300, Q5301, Q5302, Q5310, Q5311, Q53111, Q53112, Q5312, Q5313, Q5320, Q5321, Q53211, Q53212, Q5322, Q5323, Q539, Q540, Q541, Q542 Q543, Q544, Q548, Q549, Q550, Q551, Q5520, Q5521, Q5522, Q5523, Q5529, Q554, Q555, Q5561, Q5562, Q5563, Q5564, Q5569, Q558, Q559, Q562, Q563, Q564 |
| Congenital Malformations of the Urinary System | Q600, Q601, Q602, Q603, Q604, Q605, Q606, Q6100, Q6101, Q6102, Q6111, Q6119, Q612, Q613, Q614, Q615, Q618, Q619, Q620, Q6210, Q6211, Q6212, Q622, Q6231, Q6232, Q6239, Q624, Q625, Q6260, Q6261, Q6262, Q6263, Q6269, Q627, Q628, Q630, Q631, Q632, Q633, Q638, Q639, Q640, Q6410, Q6412, Q6419, Q642, Q6431, Q6432, Q6433, Q6439, Q644, Q645, Q646, Q6470, Q6471, Q6473, Q6474, Q6479, Q648, Q649 |
| Congenital Malformations and Deformations of the Musculoskeletal System | Q6500, Q6501, Q6502, Q651, Q652, Q6530, Q6531, Q6532, Q654, Q655, Q656, Q6581, Q6582, Q6589, Q659, Q660, Q6600, Q6601, Q6602, Q661, Q6611, Q6612, Q662, Q6621, Q6622, Q66221, Q66222, Q66229, Q663, Q6630, Q6631, Q6632, Q664, Q6640, Q6641 Q6642, Q6650, Q6651, Q6652, Q666, Q667, Q6670, Q6671, Q6672, Q6680, Q6681, Q6682, Q6689, Q669, Q6690, Q6691, Q6692, Q670, Q671, Q672, Q673, Q674, Q675, Q676, Q677, Q678, Q680, Q681, Q682, Q683, Q684, Q685, Q686, Q688, Q690, Q691, Q692, Q699, Q7000, Q7001, Q7002, Q7003, Q7010, Q7011, Q7012, Q7013, Q7020, Q7021, Q7022, Q7023, Q7030, Q7031, Q7032, Q7033, Q704, Q709, Q7101, Q7102, Q7111, Q7112, Q7113, Q7121, Q7122, Q7123, Q7130, Q7131, Q7132, Q7133, Q7140, Q7141, Q7142, Q7143, Q7150, Q7151, Q7152, Q7153, Q7160, Q7161, Q7162, Q7163, Q71811, Q71812, Q71813, Q71819, Q71891, Q71892, Q71893, Q71899, Q7190, Q7191, Q7192, Q7193, Q7200, Q7201, Q7202, Q7203, Q7213, Q7220, Q7222, Q7230, Q7231, Q7232, Q7233, Q7240, Q7241, Q7242, Q7243, Q7251, Q7253, Q7260, Q7261, Q7262, Q7263, Q7270, Q7271, Q7272, Q7273, Q72811, Q72812, Q72813, Q72819, Q72891, Q72892, Q72893, Q72899, Q7290, Q7291, Q7292, Q7293, Q730, Q731, Q738, Q740, Q741, Q742, Q743, Q748, Q749, Q750, Q751, Q752, Q753, Q754, Q758, Q759, Q760, Q761, Q763, Q76412, Q76413, Q76414, Q76415, Q76419, Q76425, Q76427, Q76428, Q76429, Q7649, Q765, Q766, Q767, Q768, Q769, Q770, Q771, Q772, Q773, Q774, Q775, Q776, Q777, Q778, Q779, Q780, Q781, Q782, Q783, Q784, Q785, Q788, Q789, Q790, Q791, Q792, Q793, Q794, Q7951, Q7959, Q796, Q7960, Q7962, Q7963, Q798, Q799 |
| Other Congenital Malformations | Q800, Q801, Q802, Q804, Q808, Q809, Q810, Q811, Q812, Q818, Q819, Q820, Q821, Q822, Q823, Q824, Q825, Q826, Q828, Q829, Q830, Q831, Q832, Q833, Q838, Q839, Q840, Q842, Q843, Q844, Q845, Q846, Q848, Q849, Q8500, Q8501, Q8509, Q851, Q858, Q859, Q860, Q861, Q862, Q868, Q870, Q871, Q8711, Q8719, Q872, Q873, Q8740, Q87410, Q8743, Q875, Q8781, Q8782, Q8789, Q8901, Q8909, Q891, Q892, Q893, Q894, Q897, Q898, Q899 |
| Chromosomal Abnormalities, Not Elsewhere Classified | Q900, Q901, Q902, Q909, Q910, Q911, Q912, Q913, Q914, Q915, Q916, Q917, Q921, Q922, Q925, Q9261, Q927, Q928, Q929, Q931, Q933, Q934, Q935, Q9351, Q9359, Q937, Q9381, Q9382, Q9388, Q9389, Q939, Q950, Q951, Q959, Q960, Q961, Q962, Q963, Q964, Q968, Q969, Q970, Q971, Q973, Q978, Q979, Q980, Q981, Q983, Q984, Q985, Q986, Q987, Q988, Q990, Q991, Q992, Q998, Q999 |
